# Married Women Doctors and Their Appointments

**Published:** 1914-04-18

**Authors:** 


### The Hospital

The Workers' Newspaper of Administrative Medicine and Institutional Life, Administration, National Insurance and Health.

No. 1451, Vol. LVI. SATURDAY, APRIL 18, 1914. PRICE ONE PENNY.

### MARRIED WOMEN DOCTORS AND THEIR APPOINTMENTS.

As our own correspondence columns and those of the lay press have shown, a recent debate in the Council Chamber at Spring Gardens has focussed public attention upon an aspect of the work of women doctors: to wit, upon the advisability of requiring those who hold salaried posts to resign their appointments when they marry. Curiously enough, the debate aforesaid did not turn at all upon this question as one of principle; for the London County Council years ago decided that certain of its female staff—women doctors, among others—should be engaged only upon condition that marriage should entail resignation. This policy was adopted after full consideration, and has been followed ever since; and until the Council decides to abandon it, some of the members feel unable to vote for any exception in particular cases, such as the Rev. Scott Lidgett proposed at the recent meeting. The question at issue is large and complicated, and there is room for perfectly honest difference of opinion about it. It is, of course, only a particular part of the general problem of the relation of marriage and motherhood to professional work for women. With this larger question The Hospital is not concerned, except in so far as it bears upon the interests of medical women, and especially of those who are also salaried institutional or municipal officers.

Let us try to visualise first the point of view embodied in the official London County Council policy. Presumably it is thought that a medical woman during part or the whole of each pregnancy, and for some months afterwards, is not fit to carry on her work. Few doctors of either sex will dissent from this view, we imagine; and it follows that any medical woman in the Council's service will necessarily require leave of absence for periods up to eighteen months at a time on each occasion that her family is added to. Do these absences, or the care of her family in between whiles, impair her capacity for subsequent work or disorganise the department to which she belongs? Presumably it is thought that they do one or the other of these things. There are, however, married women who are not so fortunate as to have children. It may be urged that there is no need to exclude such women from their professional work. But the reply, we take it, is that if such exceptions are allowed there will be deliberate avoidance of conception by those who are not naturally sterile; that such evasions of motherhood are bad for the State and for the individuals themselves; and that no public body should even indirectly or remotely do anything to encourage them.

On the other hand, there is much that can be legitimately said for the opposite view; the two letters from medical women in our correspondence columns last week are evidence of this. It is true enough that a married woman, and *a fortiori* a mother, gains a "knowledge of life and breadth of character" (to quote one of our correspondents) which are certain to help her medical work, be it private practice or inspectional or institutional. It is undeniable that celibacy is bad for the great majority of women, and that the Council's regulations put a premium upon it. It is admittedly hard on individuals that a cast-iron rule should turn them out of their posts when for all anyone knows they may be incurably sterile. It is certainly bad for the service that feelings of injustice should pervade its members, or that they should be tempted to forego marriage rather than their profession and their remuneration.

It will be seen that the solution of this difficult problem really depends on the view taken of the larger question of women's work in general. For maternity interferes with the continuity, and therefore with the value, of women's work in other professions besides that of medicine. An excellent test of the suitability of any particular avocation for women can be based very largely upon this point. If recurring periods of non-employment, lasting for many months, are likely to impair greatly the usefulness of an individual in any given profession, then married women are not suited to that profession. This test does not exclude women from lecturing appointments under the London County Council; for these lecture tours last as a rule only three months at a time, and can be resumed with complete efficiency after prolonged absence. There is thus nothing inconsistent in the

Council's attitude to lecturers, as opposed to inspectors. Nor does it exclude women from private practice; for there are many married women doctors who give up their work when necessary, and resume it as soon as they are set free from the ties which motherhood entails.

The County Council debate, inconclusive as it was, will have done good if it results in redirecting attention to first principles in connection with women's work. The plain fact is that the medical profession has never made its voice sufficiently heard over this topic of maternity in relation to professional employment in general; and it is distinctly odd that the ventilation which it is now receiving should occur over certain appointments in the Council's medical service. Whether that body will consider again the principle which it adopted six or seven years ago we do not know; but if it does so, we trust that an attempt will be made to get to basic principles and that the subject will be considered from every possible point of view, including the medical.

